# Deep Tissue Characterization with Optical Coherence Elastography: A Comparison of Different Methods

**DOI:** 10.3390/ma15238558

**Published:** 2022-12-01

**Authors:** Asha Parmar, Gargi Sharma, Andreas Ramming, Kanwarpal Singh

**Affiliations:** 1 Max Planck Institute for the Science of Light, 91058 Erlangen, Germany; 2 Max-Planck-Zentrum für Physik und Medizin, 91054 Erlangen, Germany; 3 Department of Physics, Friedrich-Alexander-University Erlangen-Nürnberg, 91058 Erlangen, Germany; 4 Department of Medicine 1, Universitätsklinikum Erlangen, Friedrich-Alexander-University Erlangen-Nürnberg, 91054 Erlangen, Germany; 5 Department of Medicine 3, Universitätsklinikum Erlangen, Friedrich-Alexander-University Erlangen-Nürnberg, 91054 Erlangen, Germany

**Keywords:** optical coherence elastography, handheld probe, systemic sclerosis, common path OCT

## Abstract

The measurement of the biomechanical properties of the skin is of great interest since these properties play an important role in the development of several diseases such as skin cancer and systemic sclerosis. In this direction, several diagnostic tools have been developed to analyze the mechanical properties of the skin. Optical coherence elastography (OCE) is one of the emerging imaging techniques used for the characterization of the mechanical properties of the tissue quantitatively. In systemic sclerosis patients, the measurement of the mechanical properties of the deeper skin layers is desirable compared to the superficial layers. There are several variants of OCE that exist, but it is still not clear which method is more suitable for the measurement of the mechanical properties of the deeper tissue. In this work, we tested three common methods, the pulsed excitation method, the continuous wave excitation method, and the resonant frequency method, for the measurement of the mechanical properties of the deeper layers in the tissue. We found out that the pulsed wave excitation method provides the most reliable measurements in the shortest possible time compared to the other two methods.

## 1. Introduction

The biomechanical properties of tissue or cells can reveal the severity of diseases in different organs. Biomechanical properties have been associated with tumor growth [1], corneal keratoconus [2], atherosclerosis in arteries [3], and systemic sclerosis in the skin [4]. Systematic sclerosis is an autoimmune disease that leads to the thickening and hardening of the organ tissue due to an excessive accumulation of fibrous connective tissue. The biomechanical properties of the organ tissue are dramatically altered in systemic sclerosis, which can hamper the normal functioning of the internal organs, leading to organ failure. A standard method for the assessment of systematic sclerosis is to estimate the skin’s hardness or softness through the palpation method [5]. However, such an assessment is highly dependent on the experience of the clinician and can vary from person to person. To reduce inter-person variability, several techniques have been developed to either quantify the stiffness of the tissue or increase the local contrast of the diseased tissue compared to its surroundings.

Techniques such as ultrasonography [6] and magnetic resonance elastography [7] can provide tissue mechanical properties at the tissue level, whereas atomic force microscopy [8] and traction force microscopy [9] can be used to study cellular mechanical properties. Optical coherence elastography (OCE) is a relatively new technique that can assess the mechanical properties such as elasticity and stiffness of the tissue [10,11].

The application of OCE has already been demonstrated in ophthalmology [12,13] and in dermatology [14] to measure these tissues’ mechanical properties. In ophthalmology, OCE has been used to assess the stiffness of the cornea, and the retina to diagnose ocular diseases. In dermatology, OCE has been used to quantify the stiffness of the skin and diagnose diseases such as scleroderma and cancer at an early stage. Additionally, OCE has been used as a diagnostic tool in the assessment of liver fibrosis and breast cancer [15].

OCE has been performed using static/quasi-static displacement or dynamic displacement methods for quantitative and qualitative quantification of tissue elasticity [16,17,18,19]. In static/quasi-static OCE, the contact-based compression method has been used to calculate the tissue displacement on the application of the mechanical load on the tissue. The stress and strain ratio towards the applied load direction provides rapid 3D elastography images of the tissue. However, such methods are slow and susceptible to errors because of tissue movements.

Dynamic OCE is a wave-based method with high displacement sensitivity. It is advantageous in clinical applications for the viscoelastic assessment of tissues because of its simple implementation. In wave-based methods, air puff [20,21], pulsed laser [22,23], and electric piezo-transducer [24,25,26] have been used to generate vibrations or mechanical waves in the tissue. The displacement caused by the mechanical waves in the tissue is detected through the phase change measurement by the OCE system. Several different modes of mechanical excitation have been reported. These include pulsed excitation, continuous wave excitation and resonant wave excitation. The pulsed excitation can be achieved with the use of a piezo transducer, a pulsed laser, or an air puff. With a short excitation pulse, tissue dynamics at a wide frequency range can be studied. In the continuous wave excitation method, the tissue response is measured at a single frequency at a time. The frequency of the excitation wave is changed over time in order to measure the tissue response at different frequencies. Usingthe resonant frequency method, the mechanical waves in the tissue can be excited through a pulsed wave or continuous wave. After the excitation, the resonant frequency of the tissue is determined, which indicates the mechanical properties of the tissue.

Depending on the application, one or the other method can be a suitable choice to measure the tissue’s mechanical properties. Recently, a comparison study describing different strategies for a pulsed excitation method was reported for a mono-layered tissue phantom [27]. Within certain diseases such as systemic sclerosis, it is highly desirable to measure the mechanical properties of the deeper skin layers [6]. By doing so, one can minimize the effect of external factors thataffect the external skin layers the most. With all these methods available, it becomes important to identify the method thatwould provide the most reliable measurement of the deeper skin layers.

In this paper, we have developed a handheld common-path probe-based portable spectral domain-Optical coherence elastography (SD-OCE) system. Using a piezoelectric transducer, we measured the elastic properties of a multilayered tissue phantom using three different wave excitation methods. The results were compared to determine the method thatprovides the most reliable and repeatable measurement of the mechanical properties of the deeper tissue layers.

## 2. Materials and Methods

### 2.1. System Design

**Figure 1:**
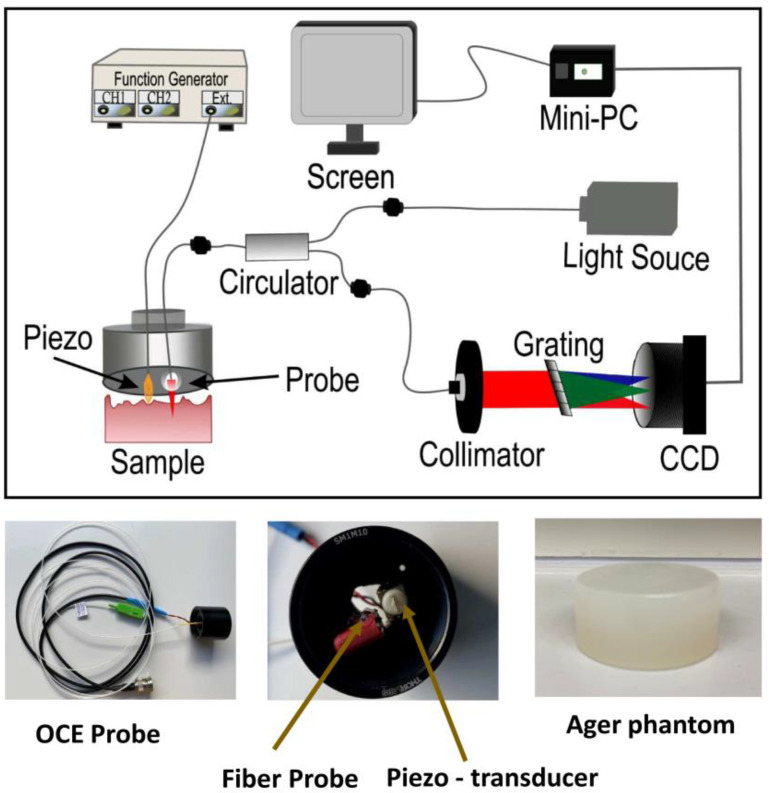
Schematic of the SD-OCE system. The lower panel on the left shows the fiber-based probe, the middle shows the tip of the probe with optical fiber and piezoelectric transducer, and the right panel shows one of the ager phantoms.

The schematic of the SD-OCE system is shown in Figure 1. In the system, we used a super luminescent diode (SLD-371, Superlum, Carrigtohill, Ireland) as a light source with a central wavelength of 840 nm and full-width half maxima bandwidth of 52 nm. The light from the SLD was coupled to a circulator (850 nm SM Circulator, PM-Optics, Burlington, MA, USA). The light from the circulator was coupled to the fiber-based probe, which consisted of a single-mode optical fiber (HI 780, AFW Technologies, Hallam, VIC, Australia) spliced to a 200 µm spacer (FG105LCA, Thorlabs, Newton, NJ, USA) and 200 µm graded index (GRIN) fiber (F-MLD, Newport Corporation, Irvine, CA, USA). The handheld OCE probe was used in a common path configuration, where the reflection from the tip of the GRIN fiber was used as a reference signal. A piezoelectric transducer (PK2FMP1, Thorlabs) was placed at a distance of 5 mm from the beam focus on the sample and used to excite the mechanical waves in the sample.The sample was illuminated with 12 mW of optical power. The light reflected from the sample was collected back by the optical fiber probe and directed towards a homemade spectrometer. The spectrometer consisted of a 1200 lines/mm diffraction grating (WP-1200/840), 80 mm focal length lens, and a 2048-pixel line scan camera (RAL2048, Basler AG, Ahrensburg, Germany) operating at 36,000-line scans per second. The images were acquired in M-mode and each M-mode scan consisted of 3600 A-scans. The interferograms from the camera were transferred to a host personal computer (NUC10i7FN-Kit- Mini-PC) via Ethernet cable. The interferograms were recalibrated from the wavelength domain to the frequency domain using a previously reported method [28]. The transfer data on the host computer was processed using a custom-designed LabView 2020 software. The mechanical wave excitation was synchronized with camera frame acquisition.

### 2.2. Sample Preparation

We prepared different stiffnessesof ager phantom with 1%, 2% and 3% concentrations and added a few drops of milk to increase the scattering of light. The thickness of the sample was approximately 10 mm. We used ager as a tissue phantom because the mechanical properties of the ager phantom mimic human tissue [29].

The developed system was tested on ager phantoms and the hand skin of a healthy volunteer. All the methods carried out in this work are in accordance with relevant guidelines and regulations from the local institutional review board (Ethikkommission der Friedrich-Alexander-Universität, Erlangen, Germany). Testing on the hand was performed as a self-test on the authors of the manuscript who signed informed consent to participate. No other human experiments were performed in this work.

## 3. Results

### 3.1. Displacement Stability

For the characterization of the developed system, we measured the displacement sensitivity of the SD-OCE system. For this, we used a glass plate as a sample and measured the displacement of the two surfaces of the glass plate with respect to the tip of the common path fiber probe. The displacement values of the first glass plate surface were subtracted from the displacement values of the second glass plate surface, which gives the displacement sensitivity of the system. The calculated displacement sensitivity of the common path system was measured to be 0.13 nm.

### 3.2. Measurement of the Mechanical Properties of Ager Phantoms and Human Skin by Pulsed Excitation Method

We used a pulsed electric signal with 1% duty cycle at 10 Hz to drive the piezoelectric transducer. When in contact with the sample, the piezoelectric transducer excites the surface acoustic waves on the sample. Surface acoustic waves are uniformly distributed on the surface of the sample, which can be used to detect the mechanical properties of the tissue. We first measured the excitation pulse directly on the surface of the piezo, which was used as a reference pulse against which all other measurements were performed. In order to measure the phase delay between the excitation pulse and the pulse at the measurement site, a cross-power spectrum fast Fourier transform (FFT) was performed between the reference pulse and the measured pulse. The phase delay was extracted from the cross-power spectrum FFT. The velocity of the mechanical waves at different frequencies was calculated using the relation [24]

(1) $$V=\frac{\left(x_{1}-x_{2}\right)\;2\pi \;f\;}{∆\varphi }.$$

where $\left(x_{1}-x_{2}\right)$ is the distance between the excitation point and the measurement point, *f* is the frequency and ∆*φ* is the phase difference between the excitation pulse and the measurement pulse at frequency *f*.

Furthermore, the sample depth probed by a certain frequency can be estimated using the relation

(2) $$z=\frac{v}{f}$$

where *z* is the depth of the sample, *v* is the velocity of the wave, and *f* is the frequency at which the sample is being probed.

**Figure 2:**
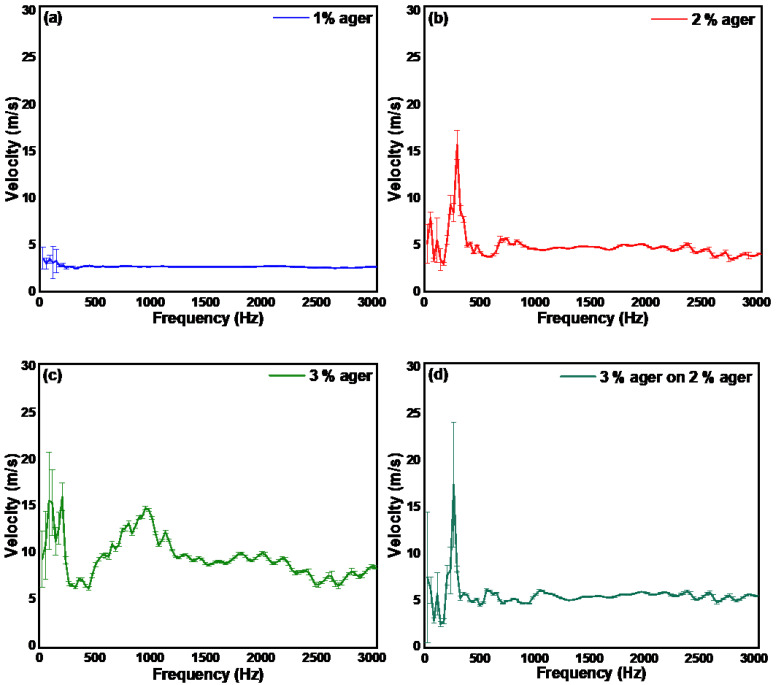
Surface acoustic wave velocity measured using pulsed excitation method in (**a**) monolayer 1%, (**b**) monolayer 2% (**c**) monolayer 3%, and (**d**) dual layer 3% on 2% ager phantom.

In Figure 2a–c, we show the velocity of the mechanical waves in the agerin response to the applied excitation wave. The calculated averaged elastic velocity of 10 mm thick, 1%, 2%, and 3% ager was found to be 3.5 m/s, 5 m/s, and 10 m/s, respectively, which corresponds to Young’s modulus of 38.95 kPa, 79.50kPa, and 318 kPa, respectively. The Young’s modulus was calculated using the relation:

(3) $$E=3\rho c_{S}^{2}$$

where ρ is the material density and $c_{s}$ is the surface acoustic wave velocity in the sample. In order to mimic a multilayered sample such as skin, we prepared a thin slice (200 µm) of 3% ager and placed it on a 10 mm thick 2% ager. In Figure 2d, we show the velocity of the mechanical waves in a multi-layered ager phantom (3% on 2%) ager. The measured velocity was found to be similar to the monolayered 2% ager. This suggests that the velocity measured in the frequency range below 3000 Hz using a pulsed excitation method represents the velocity of the deeper layers rather than the surface layers.

**Figure 3:**
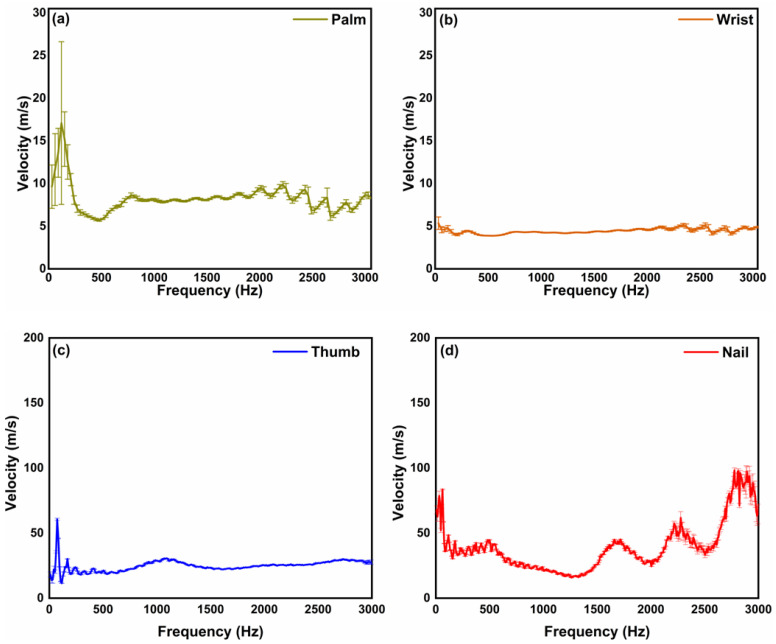
Surface acoustic wave velocity measured on (**a**) palm, (**b**) wrist, (**c**) thumb, and (**d**) nail of a healthy volunteer.

In Figure 3, we show the velocity of the mechanical waves measured on palm, wrist, thumb, and nail in a healthy volunteer. The calculated elastic velocity on the palm, wrist, thumb, and nail was found to be 7.5 m/s, 5 m/s, 24.8 m/s, and 32.9 m/s, respectively. An interesting observation from the results is that the acoustic wave velocity for the palm, wrist, and thumb is approximately the same for all the frequencies, but for the nail, the acoustic wave velocity is lower at lower frequencies and higher at higher frequencies. Since higher frequencies travel within the superficial layers of the tissue, the results for the nail suggest that the superficial layer is harder than the deeper layers, which is true in the case of the nail. The obtained results from phantoms and skin are found to be comparable with previous works.

We have also provided the measured excitation pulse from the piezo and the measured surface acoustic wave from the sample as Supplementary Figures.

### 3.3. Measurement of the Mechanical Properties of Ager Phantoms and Human Skin by Continuous Wave Excitation Method

**Figure 4:**
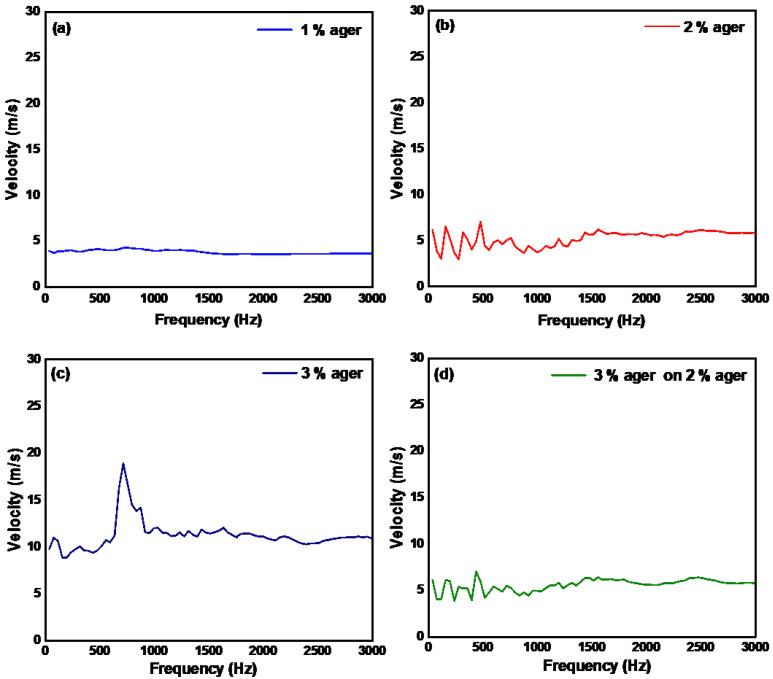
Surface acoustic wave velocity measured using continuous sine wave excitation method in (**a**) monolayer 1%, (**b**) monolayer 2%, (**c**) monolayer 3%, and (**d**) dual layer 3% on 2% ager phantom.

We used a sine wave electric signal to derive an electric piezo transducer to generate a continuous wave in the sample at frequencies ranging from 100 to 3000 Hz. In Figure 4a–d, we show the velocity of the mechanical waves in ager in response to the applied excitation wave. In order to calculate the velocity of the continuous waves on the sample, we first measured the excitation wave directly on the piezo and then on the sample. A phase delay between these two waves was calculated by performing cross-power spectrum transform on the two waves. Care must be taken since a large phase delay (greater than 180°) between the two waves will produce ambiguous results related to phase unwrapping. The phase unwrapping errors will become more prominent at higher frequencies. The calculated averaged elastic velocity of the 1%, 2%, 3%, and 3% ager on 2%ager was found to be this 3.69 m/s, 5.22 m/s, 11.18 m/s, and 5.58 m/s, respectively which corresponds to Young’s modulus of 23.01 kPa, 86.64 kPa, 397.47 kPa and 99.01 kPa, respectively.

From these measurements, we see that the obtained results are similar to the pulsed wave method, but require frequency sweeping, making it slower than the pulsed excitation method. Furthermore, the initial excitation frequency should be sufficiently low to remove the phase ambiguity due to phase unwrapping problems. For instance, in soft tissue, if we consider a velocity of 3 mm/s then, as per Equation (1), the phase ambiguity will occur at a phase difference of π radians, which translates to a frequency of 300 Hz. Thus, to detect this phase jump, the starting excitation frequency should be below 300 Hz, which would allow unwrapping the phase through unwrapping algorithms in post-processing.

### 3.4. Measurement of Depth Elasticity by Resonant Frequency Method

**Figure 5:**
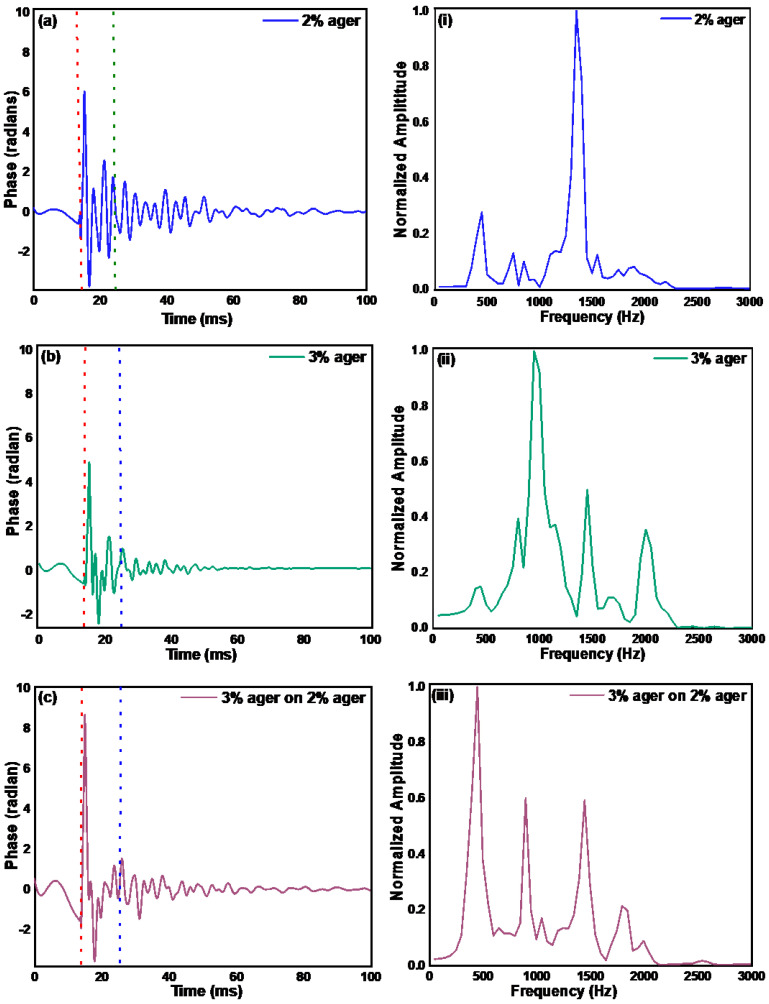
Displacement in terms of phase measured using pulsed excitation in (**a**) monolayer 2%, (**b**) monolayer 3%, and (**c**) dual layer 3% on 2% ager phantom. The first dotted red line represents the start of the excitation pulse and the blue dotted line represents the starting time from where the data were considered for calculation of the resonant frequency. The spectrum of the data after the blue dotted line in (**a**–**c**) is shown in (**i**–**iii**), respectively.

We again used the pulsed wave excitation method to generate the surface acoustic wave on the sample to measure the elastic velocity with the resonant frequency method. The natural resonant frequency of the tissue is a function of the elastic modulus of the sample. In this method, tissue is excited with a pulsed signal, and then the vibrations in the tissue are measured once the main excitation pulse has passed away. In our study, we measured the tissue vibration after a delay of 10 ms of the excitation signal and then performed FFT on the data to acquire the spectrum of the resonant frequencies. In Figure 5a–c, we show the measured phase where the first dotted line from the left represents the excitation time, and the second dotted line represents the starting time, after which the data for the resonant frequency calculation weretaken. The spectrum of the measured phase after the second dotted line is shown in Figure 5i–iii.

**Figure 6:**
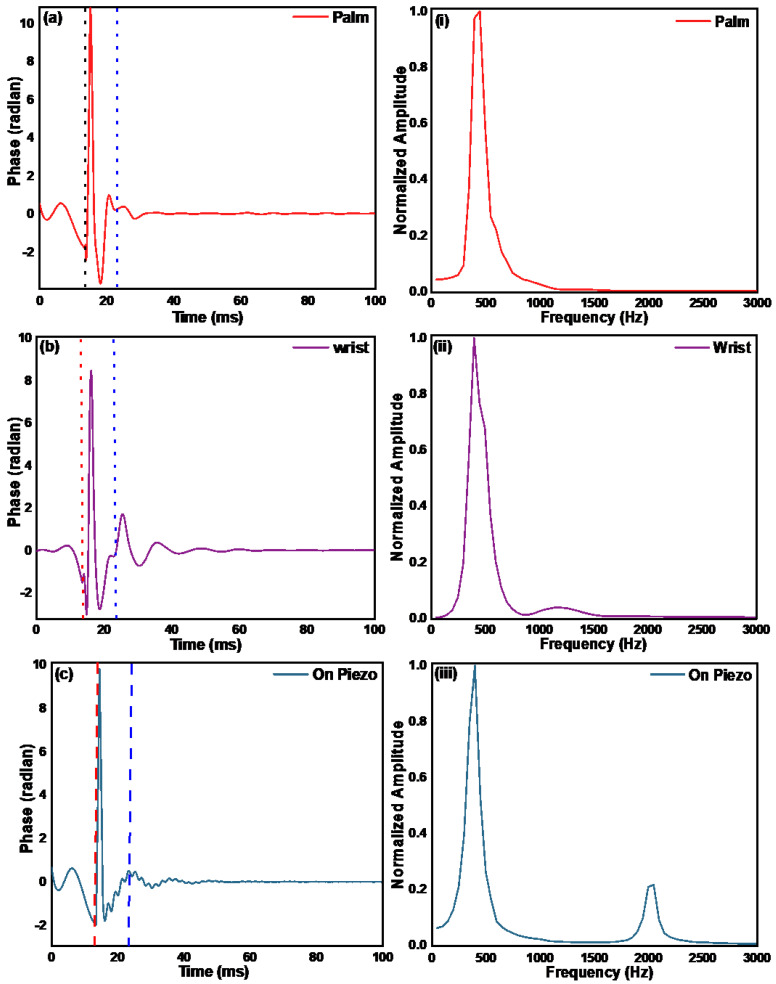
Displacement in terms of phase measured using pulsed excitation on (**a**) palm, (**b**) wrist, and (**c**) directly on piezo. The first dotted red line represents the start of the excitation pulse, and the blue dotted line represents the starting time from where the data were considered for calculation of the resonant frequency. The spectrum of the data after the blue dotted line in (**a**–**c**) is shown in (**i**–**iii**), respectively.

In order to calculate Young’s modulus from the resonant frequencies, one needs to calibrate the system, but from the resonant frequency spectrum in Figure 5, one can see that it is difficult to assign a single resonant frequency to the sample. Moreover, the resonant frequency spectrum of the 3% over 2% ager phantom is very different from that ofthe 2% ager, which suggests that the resonant frequency method does not provide a reliable measurement of the mechanical properties of deeper layers of the sample. Furthermore, we measured the resonant frequencies in the skin at the palm and wrist, as shown in Figure 6a–c, and we did not see any resonant frequencies, which could be because of the fact that the resonant frequencies are highly dependent on the boundary conditions of the sample. The resonant frequency seen in the palm and wrist data in Figure 6 is due to the piezo and is not related to the skin properties. This is verified when we look at the resonant frequency data for the piezo pulse only. These measurements suggest that the resonant frequency method might not be a suitable choice for measuring the mechanical properties of deeper skin layers.

## 4. Conclusions

We have developed a portable system with an SD-OCE common-path probe approach for the assessment of the mechanical properties of deeper tissue layers for three different excitation mechanisms. We used a piezo-transducer and a single-mode optical fiber-based common path probe to generate and detect surface acoustic waves, respectively. Although a piezoelectric transducer was used to excite the surface acoustic waves in the sample, the methodology adopted in this work is compatible with other modes of excitation such as acoustic radiation force and pulsed laser. However, we would like to point out that the excited frequencies themselves might be different for different excitation methods depending on the excitation pulse width. By measuring the displacement between the adjacent A-scans in M mode, we calculated the surface acoustic wave velocity traveling on the tissue surface caused by the excitation wave. Due to the common path approach and SD-OCE system, the undesired optical path difference changes between the reference signal and the sample signal were minimized. The developed system is capable of measuring extremely small movements (sub-nanometers) of the tissue that were caused by the excitation wave. We used three different mechanisms to measure the tissue properties, i.e., pulsed wave excitation, continuous wave excitation, and resonant frequency method. We tested these approaches using tissue phantoms made of ager, which is known to possess mechanical properties similar to soft biological tissue. It would have been meaningful to compare the results obtained for ager phantoms with some standard method such as a rheometer, but since such devices cannot provide elasticity measurement for multilayered samples, it would be difficult to compare the results directly.Through these measurements, we found out that the pulsed wave excitation method provides a reliable and repeatable measurement of the mechanical properties of deeper tissue layers. The fact that the pulsed wave excitation method provides access to a large frequency band at which the sample can be studied, it can be used to measure the elastic properties of multilayered samples by measuring the phase velocities at different frequency bands. A certain frequency band then can be associated with a certain depth using Equation (2). In our results for 3% ager over 2% ager phantom, we could only measure the acoustic velocities for 2% ager and not for the 3% because the 3% ager layer was only 200 µm. To probe such a thin layer, we would have required excitation frequencies above 30 kHz, but our system provided a reliable spectrum only up to 3 kHz, which limits our probing depth to approximately 1 mm and above. Nevertheless, a properly designed piezo or a nanosecond laser with an excitation pulse width in a nanosecond regime should be able to achieve higher excitation frequencies. The continuous wave excitation method provides similar measurement to the pulsed wave excitation method but suffers from phase ambiguity error and long acquisition time. The resonant frequency method did not provide a reliable measurement of the mechanical properties of deeper layers of the sample. Our current study suggests that the pulsed wave excitation method might be a better choice to measure the mechanical properties of deeper layers in the tissue. This study will be helpful in diseases, such as systemic sclerosis, where the measurement of the mechanical properties of the deeper layers of the skin is desirable.

## Data Availability

The data that support the findings of this study are available from the corresponding author upon reasonable request.

## Supplementary Materials

The following supporting information can be downloaded at: https://www.mdpi.com/article/10.3390/ma15238558/s1, Figure S1: Excitation pulse from the piezo and the surface acoustic wave pulse measured on the sample using pulsed excitation method in (a) monolayer 1%, (b) monolayer 2% (c) monolayer 3% and (d) dual layer 3% on 2 % ager phantom; Figure S2: Excitation pulse from the piezo and the surface acoustic wave pulse measured on (a) palm (b) wrist (c) thumb and (d) nail of a healthy volunteer.
