# Supplementary material for: Deep Tissue Characterization with Optical Coherence Elastography: A Comparison of Different Methods
Source: Materials (Basel). 2022 Dec 1;15(23):8558. doi: 10.3390/ma15238558 (PMC9740038; doi:10.3390/ma15238558)
Supplement: Supplementary file 1 [file materials-15-08558-s001.zip › materials-1954560-supplementary.pdf]

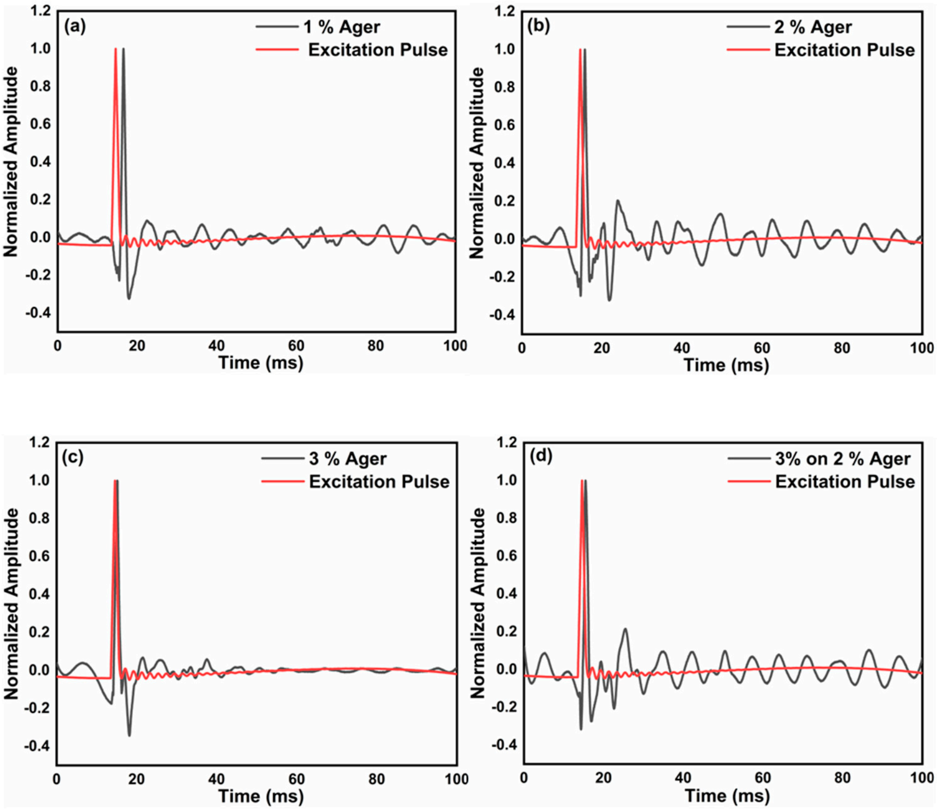

Supplementary Figure S1. Excitation pulse from the piezo and the surface acoustic wave pulse measured on the sample using pulsed excitation method in (a) monolayer 1%, (b) monolayer 2% (c) monolayer 3% and (d) dual layer 3% on 2 % ager phantom.

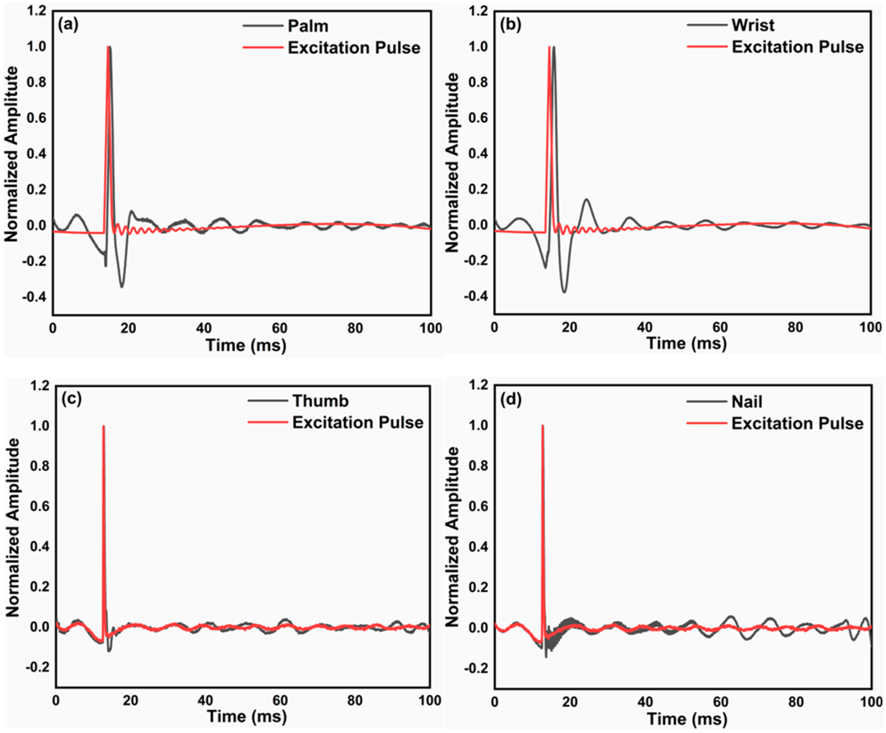

Figure S2. Excitation pulse from the piezo and the surface acoustic wave pulse measured on (a) palm (b) wrist (c) thumb and (d) nail of a healthy volunteer.
